# Systemization of a pharmacy technician career ladder in a multi-hospital system

**DOI:** 10.1016/j.rcsop.2021.100036

**Published:** 2021-06-18

**Authors:** Niaz Deyhim, Sunny B. Bhakta, Alex C. Varkey, Daniel L. Metzen, Divya Varkey, René J. Martinez, Kevin W. Garey

**Affiliations:** a Houston Methodist Hospital, 6565 Fannin Street, Houston, TX 77030, United States of America; b University of Houston, College of Pharmacy, 4849 Calhoun Road, Houston, TX 77204, United States of America

**Keywords:** Pharmacy technician, Career ladder, Pharmacy promotion, Human resources

## Abstract

**Purpose:**

Hospital consolidation into larger, systemized health systems has enabled system-wide standardization of promotion processes, including pharmacy technician career ladders. However, whether system standardization affects the job satisfaction or outcomes of pharmacy technicians is unknown. The purpose of this project was to assess pharmacy technician perceptions and outcomes after systemization of a pharmacy technician career ladder.

**Methods:**

Pharmacy technician satisfaction scores and outcomes (promotion and turnover rates) were assessed in an eight-hospital health system before and after systemization of a pharmacy technician career ladder.

**Results:**

Two hundred and forty-nine pharmacy technicians were employed during the pre-intervention (*n* = 104) and post-intervention (*n* = 145) time periods. One hundred and twenty-three of 145 (84.83%) pharmacy technicians completed a job satisfaction survey after implementation of the system-wide technician career ladder. Overall satisfaction for the career ladder averaged 3.8 ± 0.61 or between neutral to positive satisfaction. There was no difference in total satisfaction regardless of teaching (3.8 ± 0.59) or community hospital (3.8 ± 0.63) location (*p* = 0.53) or stratifying by Pharmacy Technician status. A total of 50 pharmacy technicians were hired during the study period, either during the pre-implementation (*n* = 36) or post-implementation (*n* = 14) time periods. Time to the first promotion averaged 1.73 ± 1.00 years in the pre-implementation period and 1.36 ± 0.55 years in the post-implementation period (*p* = 0.20). Technician voluntary turnover was similar between the time periods.

**Conclusion:**

In conclusion, the standardization of a systems-level pharmacy technician promotion ladder from a single hospital to a systems-level was associated with positive job satisfaction and similar promotions and turnover rates as the historic, single hospital-based promotion ladder.

## Highlights

• Healthcare systemization has necessitated systemization of many processes including pharmacy technician career ladders.
• This project assessed pharmacy technician perceptions and outcomes after systemization of a pharmacy technician career ladder.
• Systems-level pharmacy technician promotion ladder was associated with positive job satisfaction and similar promotions and turnover rates.

## 1 Introduction

Pharmacy technicians perform essential roles helping to maintain effective, quality operations within a pharmacy department.^1^, ^2^, ^3^, 4. The evolving pharmacy practice model toward increased patient care has necessitated that pharmacy technicians evolve to provide enhanced support for non-clinical duties.^2^ Tech-check-tech programs are a notable example of the advancing role of pharmacy technicians to provide innovative functions that allow pharmacists to provide enhanced patient care by decreasing the time needed for distributive oversight.^5^ These advanced functions have allowed for the creation of pharmacy technician career ladders to motivate pharmacy technicians to pursue professional development and cultivate advanced job functions and leadership skills.^2^, ^3^, 4.^,^6., 7, 8 Pharmacy technician career ladders have shown other positive attributes, including reduced pharmacy technician turnover and more positive views of salary, coworker relationships, and resource utilization.^9^ This increased job satisfaction then leads to further strengthening advanced pharmacy practice models.^10^

Most pharmacy technician career ladders are developed and standardized to a single hospital to maximize the needs of each hospital's unique systems. However, consolidation of the healthcare system has increased the need for standardized procedures amongst hospitals, including pharmacy technician career ladders.^11^ The positive effects of pharmacy technician career ladders may be tempered by the potential loss of autonomy and erosion of professional benefits gained by a career ladder that is uniquely tailored to an individual hospital.^12^ Houston Methodist is an eight-hospital nonprofit health system that consists of an academic medical center located in the Texas Medical Center and seven community hospitals within the Greater Houston Area. For many years, each hospital had maintained its own unique pharmacy technician career ladder pathway. However, the systemization of Houston Methodist necessitated a systems-level pharmacy technician career ladder. This provided a unique opportunity to understand better how to develop a standardized pharmacy technician career ladder across a large and diverse health system. The objective of this study was to describe a standardized, health-systems approach for a pharmacy technician career ladder and assess pharmacy technician attitudes and outcomes after the systemization of an existing career ladder.

## 2 Methods

### 2.1 Study design and setting

Houston Methodist pharmacy consists of 319 pharmacists and 273 pharmacy technicians across eight hospitals. The standardized pharmacy technician career ladder plan was finalized in April 2015 with a three-year rollout plan. The revised job descriptions were approved in November 2016 with full implementation of the pharmacy technician career ladder starting in January 2017. For this project, the periods after full implementation (January 2017–December 2019) were compared to a time period before starting the intervention (January 2013–December 2015). This study was approved by the Houston Methodist Research Institute Institutional Review Board (IRB) as a quality-improvement initiative exempt from IRB approval.

### 2.2 Creation and description of the systemized pharmacy technician career ladder

The vision for the system-wide pharmacy technician career ladder was to provide standardized job descriptions and promotion pathways across the health system without compromising the benefits observed from prior ladders. The implementation of the pharmacy technician career ladder occurred over three years (2015–2018). Stage one was to identify inconsistencies between job descriptions and job titles at each hospital and requirements for promotion. The task was coordinated by the Houston Methodist System Pharmacy Council, consisting of hospital directors of pharmacy within the health system. The Council reviewed facility-specific pharmacy technician job descriptions for each career ladder level and consolidated them into singular system-wide descriptions for each level. The consolidated job descriptions delineated the primary job responsibilities within the technician roles and further specified uniform experience requirements for hiring and promotion. The standardized pharmacy technician career ladder was approved by Houston Methodist System Pharmacy Council in November 2016 (Table 1).

**Table 1:** Pharmacy technician standardized career ladder job descriptions.

Characteristic	HMS^a^ Pharmacy Technician Job Level			FSR^b^ Pharmacy Technician Job Level			
	I	II	III	I	II	III	IV
High school diploma/general equivalency degree	Yes	Yes	Yes	Yes	Yes	Yes	Yes
≥2 years of college	Preferred	Preferred	Preferred	No	No	No	No
Pharmacy technician or intern license	Yes	Yes	Yes	No	Yes	Yes	Yes
ACPE^c^ intravenous certification	Yes	Yes	Yes	No	No	No	No
Tech-check-tech certification	No	No	Yes	No	No	No	No
Minimum number of years hospital experience	0–1	2	Promotion only	0	2	4	6
Above-average performance to meet promotion Criteria	No	Yes	Yes	No	Yes	Yes	Yes
Pass tech-check-tech examination	No	No	Yes	No	No	No	No
Proficient in work areas	<3	≥3	≥5	<7	≥7	≥7	≥7
Trains new staff	No	Yes	Yes	No	No	No	Yes
Participation in quality improvement projects	Yes	Yes	Yes	No	Yes	Yes	Yes
Assists with pharmacy programs/technology	No	Yes	Yes	No	No	Yes	Yes
Assists with pharmacy operations workflow	No	No	Yes	No	No	Yes	Yes

a HMS = Houston Methodist System.

b FSR = Fort Sanders Regional.

c ACPE = Accreditation Council for Pharmacy Education.

### 2.3 Pharmacy technician promotions and turnover before and after systemization of the pharmacy technician career ladder

Pharmacy technician data was obtained from the Methodist Administrative Resource System Human Resources (HR) Department. Data included institution affiliation, entry job code, hire date, promotion date, gender, and age range. Employee termination or voluntary leave dates were provided when applicable. Promotion and turnover rates for pharmacy technicians were calculated before and after systemization of the pharmacy technician career ladder.

Using concepts from the theory of reasoned action (TRA), a satisfaction questionnaire was designed to measure current employee perceptions toward a system pharmacy technician career ladder and appraise predictive intent and employee motivation for advancement (Table 2).^13^ The TRA represents a theoretical construct within social psychology to explain the specific behaviors of individuals based upon delineated motivational factors.^14^ Questionnaire domains and statements were designed to align with the TRA's key concepts: behavior, behavioral intention, attitude, behavioral belief, evaluation, subjective norm, normative beliefs, and motivation to comply. The survey was grouped into four domains: leadership and career advancement (three questions), societal expectations (three questions), experience and skill-based (two questions), and incentivized motivation (two questions). Each domain of two to three questions was first averaged to obtain the domain scores. The average of these scores was then calculated to acquire an overall satisfaction score. The survey questions were created by the principal investigator (ND) with input from pharmacy personnel involved in the pharmacy technician program. The questions were first beta-tested with senior-level pharmacy technicians or Pharmacy Technician IIIs to assure understanding of the concepts involved in each question. Then, after modifications based on feedback from the beta-testing, the survey was distributed to all technicians via a confidential Qualtrics survey.

**Table 2:** Theory of reasoned action domains with questions (all questions were answered on a 5-point Likert scale bounded by Strongly Disagree (1) to Strongly Agree (5)).

Leadership and career advancement
1. Employment at a location with potential for career advancement is important during consideration of available job opportunities.
2. A pharmacy technician career ladder motivates me to qualify for promotion.
3. I prefer an employment position that is perceived as a leadership role at my institution.
Societal expectations
4. I would like for work peers to perceive me as a responsible, trustworthy individual.
5. Most people that I work with would agree that I enjoy roles with increased responsibility and expectations.
6. It is expected of me that I participate in a pharmacy technician career ladder.
Experience and skill-based
7. Years of employment will likely contribute to promotion consideration by pharmacy management.
8. I feel that I receive adequate opportunities for training to support advancement within the pharmacy technician career ladder.
Incentivized motivation
9. Pharmacy technicians would not want to participate in a career ladder without an increase in pay rate and/or employee benefits.
10. Once I reached my desired career level, I lose motivation to progress further with responsibilities and leadership roles without an associated increase in hourly pay rate.

### 2.4 Study endpoints

The primary endpoint was to assess the perceptions of pharmacy technicians toward career advancement through the pharmacy technician satisfaction survey. Secondary endpoints pertained to pharmacy technician turnover, promotion, and voluntary leave details pre-and post-systemized career ladder implementation.

### 2.5 Data collection

The 10-question satisfaction survey was constructed with Likert scale format via an online platform and distributed to the pharmacy technician staff employed at each facility via a confidential, anonymous survey. A three-week timeframe was established for completion of the survey, open to respondents from January 24th through February 14th of 2020. Employee response confidentiality was maintained through the survey's design not to include questions that solicit employee identification.

### 2.6 Statistical analysis

The Shapiro-Wilk normality test dictated nonparametric analysis of continuous data with the Wilcoxon rank-sum test or Mann-Whitney *U* test. The chi-squared test or Fisher's exact test was utilized for the analysis of categorical variables. Statistical analyses and tests were conducted with Stata/SE (version 15.1, College Station, Texas) or SAS (version 9.4, Cary, North Carolina). A *p*-value less than 0.05 was considered significant.

## 3 Results

### 3.1 Baseline demographics

Two hundred and forty-nine pharmacy technicians were employed during the periods, including pre-intervention (*n* = 104) and post-intervention (*n* = 145) time periods. The distribution of technician levels and demographics is shown in Table 3.

**Table 3:** Pharmacy technician baseline demographics.

Characteristic, no. (%)	Pre-Intervention (*n* = 104)	Post-Intervention (*n* = 145)	P-Value
Age, years^a^			
18–25	0 (0.00)	5 (3.45)	0.07
26–35	47 (45.19)	72 (49.66)	0.49
36–40	17 (17.31)	23 (15.86)	0.92
40+	39 (37.50)	45 (31.03)	0.29
Gender			
Male	53 (33.65)	42 (28.97)	<0.001
Community institutions			
Pharmacy Technician I	24 (23.08)	28 (19.31)	0.47
Pharmacy Technician II	28 (26.92)	70 (48.28)	0.001
Pharmacy Technician III	8 (7.69)	0 (0.00)	0.001
PRN employees	17 (16.35)	0 (0.00)	<0.001
Academic medical center			
Pharmacy Technician I	22 (21.15)	43 (29.66)	0.13
Pharmacy Technician II	0 (0.00)	4 (2.76)	0.11
Pharmacy Technician III	0 (0.00)	0 (0.00)	N/A
PRN employees	5 (4.81)	0 (0.00)	0.01

a Represents age range at the time of initial hire.

### 3.2 TRA survey

One hundred and twenty-three of 145 (84.83%) pharmacy technicians completed the satisfaction survey after implementation of the system-wide technician career ladder. Responses were split evenly between the academic medical center and community hospital settings. Respondents included Pharmacy Technician I (*n* = 21; 17.07%), Pharmacy Technician II (*n* = 71; 57.72%), and Pharmacy Technician III (*n* = 31; 25.20%). Overall satisfaction for the career ladder averaged 3.8 ± 0.61, or between neutral to positive satisfaction. All domains averaged above-neutral satisfaction. Domain satisfaction was highest for societal expectations (4.12 ± 0.66), followed by leadership and career advancement (4.07 ± 0.81), experience and skill-based (3.51 ± 1.15), and incentivized motivation (3.49 ± 1.04). There was no difference in total satisfaction regardless of academic (3.8 ± 0.59) or community hospital (3.8 ± 0.63) location (*p* = 0.53) or stratifying by Pharmacy Technician I (3.84 ± 0.64), Pharmacy Technician II (3.69 ± 0.63), or Pharmacy Technician III (4.00 ± 0.50) status (*p* = 0.06). Pharmacy technician survey results were similar by technician levels for all domains, except leadership and career advancement (*p* = 0.01). Survey scores were 4.1 ± 0.85 for Pharmacy Technician I, decreased to 3.9 ± 0.84 for Pharmacy Technician II, and increased to 4.4 ± 0.60 for Pharmacy Technician III. Controlling for multiple comparisons, leadership scores were significantly higher for Pharmacy technician IIIs compared to Pharmacy technician IIs (*p* = 0.0030).

### 3.3 Career advancement and employee turnover

A total of 50 pharmacy technicians were hired during the study period, either during the pre-implementation (*n* = 36) or post-implementation (*n* = 14) time periods. The time to the first promotion averaged 1.73 ± 1.00 years in the pre-implementation period and 1.36 ± 0.55 years in the post-implementation period (*p* = 0.20). Thirteen of 50 pharmacy technicians also progressed to a second promotion to Pharmacy Technician III, 11 in the pre-implementation period and 2 in the post-implementation period. The time to the second promotion was 2.94 ± 1.00 years in the pre-implementation period and 2.01 ± 0.00 years in the post-implementation period (*p* = 0.22).

Technician voluntary turnover was similar between the two time periods. In the pre-implementation period, 9 of 36 (25.00%) hired pharmacy technicians voluntarily left their employment compared to 3 of 14 (21.43%) hired technicians during the post-implementation period (*p* = 0.76).

## 4 Discussion

The systemization of healthcare has necessitated that pharmacy technician promotion pathways developed by single hospitals also be systemized to allow for a consistent promotion pathway between the institutions. Pharmacy technician promotion pathways have been shown to reduce pharmacy technician turnover and result in more positive views of salary, coworker relationships, and resource utilization.^9^ For many years, hospitals within our health system maintained an autonomous pharmacy technician promotion pathway unique to each hospital. In 2016, we implemented a system-wide pharmacy technician promotion pathway amongst the eight hospitals within our health system. During the development of the system-wide promotion pathway, some concern was expressed whether the quality of the promotion pathway would diminish as each hospital would not have the ability to tailor the promotion pathway to the unique attributes of the individual hospital. However, the systemization allowed for more flexible use of the pharmacy technician workforce and allowed a systems-level approach to operationalizing the program. To answer this question, we assessed pharmacy technician job satisfaction after implementing a systems-level promotion pathway and promotion and voluntary pharmacy technician departures before and after implementation of the system-wide promotion pathway. The average job satisfaction scores for the new program were consistent amongst all three promotion levels of pharmacy technicians and averaged neutral satisfaction or greater. In addition, the time to promotion was comparable in the new system, and voluntary pharmacy technician departures were also similar. Thus, the rollout of the new promotion pathway did not seem to diminish the success of prior programs. Strengths of this study include a multi-year evaluation and a large number of pharmacy technicians assessed during the time period.

We were able to identify one prior study that described the development and benefits of a pharmacy technician career ladder (Table 1).^9^ The ladder was implemented at a 575-bed community hospital and consisted of four stages with specific skills required to advance to each stage. Similar to our study, pharmacy technicians expressed positive job satisfaction with implementing the ladder, and advanced technicians were assigned select tasks traditionally done by licensed pharmacists before implementation. This allowed for pharmacists to commit to enhanced clinical activities due to the time savings. Our plan incorporated many facets of pharmacy technician professional activities not available when the prior ladder was developed, such as pharmacy technician certification for sterile products. However, both models demonstrate benefit and can be used as a model for other systems or hospitals interested in developing a pharmacy technician career ladder.

This study has certain limitations. This was a non-randomized study, and thus our findings could be reflective of biases amongst pharmacy technicians employed within our health systems. Second, we were not able to survey pharmacy technicians prior to implementation of the system-wide career ladder, and it is plausible that job satisfaction may have been higher prior to implementation. Pharmacy technicians were studied for promotions and turnover during the entire study period. Thus, technicians hired at a later date had a shorter evaluation time.

## 5 Conclusion

In conclusion, the standardization of a systems-level pharmacy technician promotion ladder from a single hospital to a systems-level was associated with positive job satisfaction and similar promotions and turnover rates as the historic, single hospital-based promotion ladder. We feel this ladder could be used for other hospitals or health systems interested in developing a standardized promotion pathway.

## Funding

This research study was awarded by the American Society of Health-System Pharmacists Foundation 2019 New Practitioner Leadership Development Research Grant.

## Declaration of Competing Interest

The authors declare that they have no known competing financial interests or personal relationships that could have appeared to influence the work reported in this paper.
